# Benign peritoneal cystic mesothelioma mimicking peritoneal tuberculosis

**DOI:** 10.1002/ccr3.6672

**Published:** 2022-12-05

**Authors:** Chayma Soltani, Khalaf Ben Abdallah, Yasser Bouchallouf

**Affiliations:** ^1^ Nephrology Department University of Tunis El Manar Faculty of Medicine of Tunis Tunis Tunisia; ^2^ Gastroenterology Department University of Tunis El Manar Faculty of Medicine of Tunis Tunis Tunisia; ^3^ Radiology Department University of Tunis El Manar Faculty of Medicine of Tunis Tunis Tunisia

## Abstract

In immunodeficient patients with repetitive peritoneal aggression, cystic mesothelioma should be considered as a differential diagnosis to peritoneal tuberculosis that must be ruled out in endemic countries.

In immunodeficient patients with repetitive peritoneal aggression, cystic mesothelioma should be considered as a differential diagnosis to peritoneal tuberculosis that must be ruled out in endemic countries.

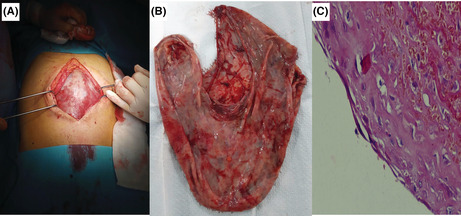

## 1 CASE SUMMARY AND DISCUSSION

**FIGURE 1:**
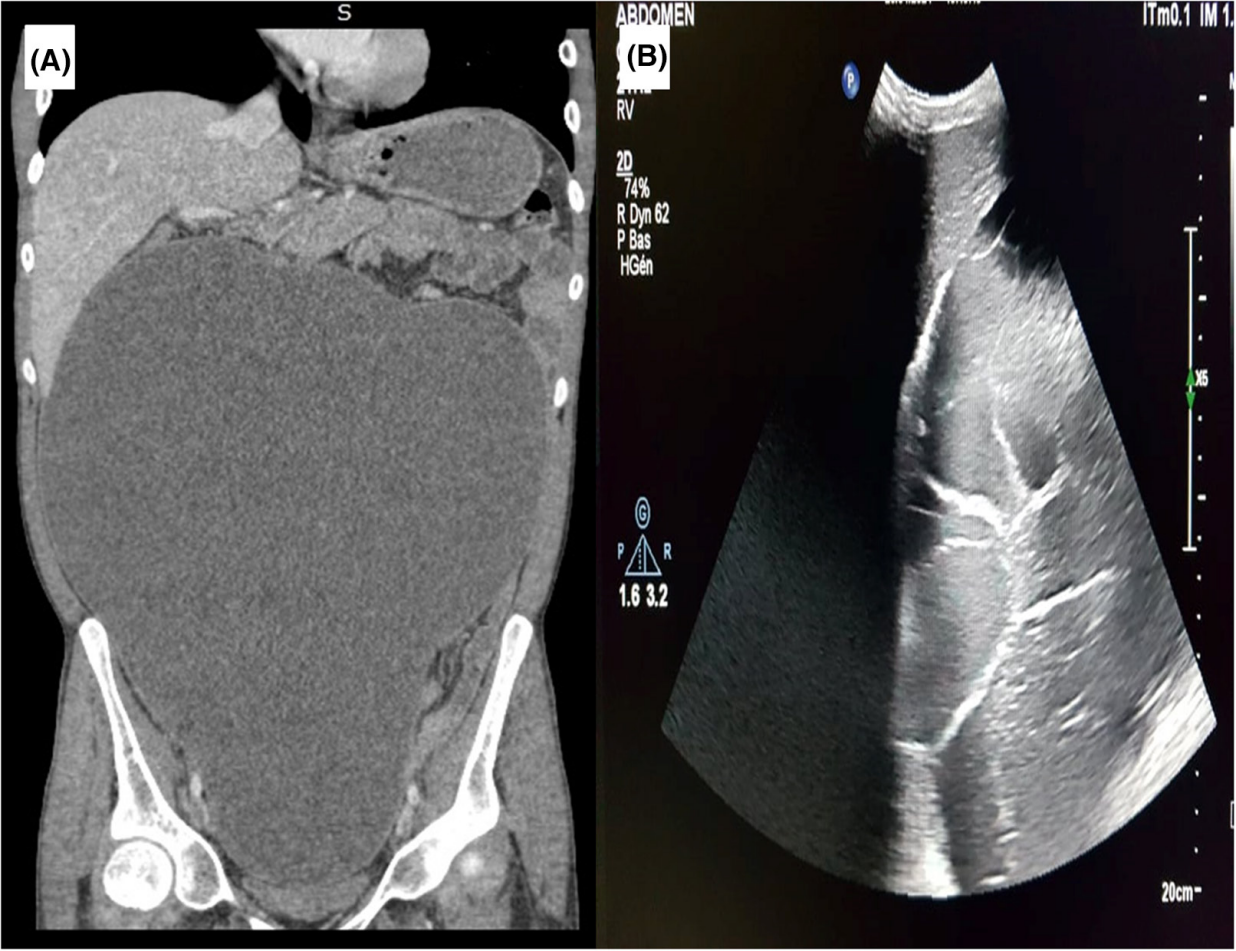
Radiological findings in abdominal ultrasound (A) and computed tomography (B): large abundance septated ascites

**FIGURE 2:**
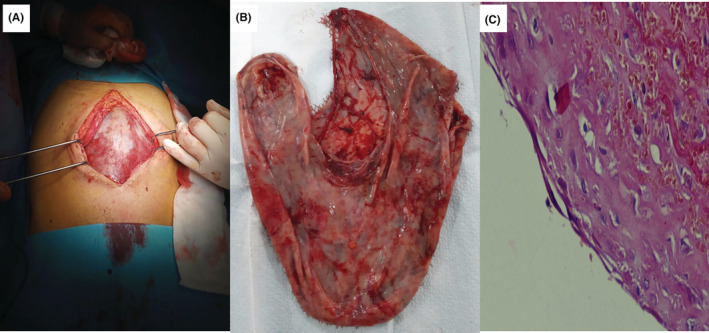
Macroscopic view of the cyst at laparotomy (A) and after surgery resection (B) and pathology showing mesothelial cyst without signs of malignancies. (C) Pathology image of the resected cyst

A 22‐year‐old male patient, with a history of end‐stage renal failure related to Alport syndrome treated with peritoneal dialysis, presented with progressive abdominal distension. Physical examination of the abdomen revealed ascites of large abundance. Laboratory tests showed elevated C‐reactive protein at 242 mg/L. Peritoneal fluid analysis revealed exudate ascites poor of cells. Abdominal ultrasound showed floating membranes, and computed tomography concluded to a huge abdominal cystic mass (Figure 1). Initially, there were no sufficient arguments supporting the diagnosis of peritoneal tuberculosis. Nevertheless, we decided to further investigate the cause of the ascites using laparotomy. At exploratory laparotomy, we discovered a cystic mass adhering to the anterior wall. We perceived with a complete cyst resection. Pathology and immunohistochemistry analysis concluded the diagnosis of cystic mesothelioma of the peritoneum without signs of malignancies. (Figure 2) Cystic mesothelioma is secondary to peritoneal aggression. Formed cyst evolves gradually by triggering continuous irritating micro‐aggressions. Malignant transformation remains exceptional.^1^ Recurrence is frequent.^2^

## AUTHOR CONTRIBUTIONS

**Chayma Soltani:** Conceptualization; investigation; writing – review and editing. **Khalaf Ben Abdallah:** Conceptualization; investigation; methodology; validation; writing – original draft; writing – review and editing. **Yasser Bouchallouf:** Conceptualization; investigation; validation; visualization.

## ACKNOWLEDGMENTS

None.

## FUNDING INFORMATION

The authors declared that no grants were involved in supporting this work.

## CONFLICT OF INTEREST

No competing interests were disclosed.

## CONSENT

Written informed consent to publish his clinical details and images was obtained from the patient.

## Data Availability

All data underlying the results are available as part of the article and no additional source data are required.
